# 25-hydroxyvitamin D3 and 1,25-dihydroxyvitamin D3 exert distinct effects on human skeletal muscle function and gene expression

**DOI:** 10.1371/journal.pone.0170665

**Published:** 2017-02-15

**Authors:** Zaki K. Hassan-Smith, Carl Jenkinson, David J. Smith, Ivan Hernandez, Stuart A. Morgan, Nicola J. Crabtree, Neil J. Gittoes, Brian G. Keevil, Paul M. Stewart, Martin Hewison

**Affiliations:** 1 Institute of Metabolism and Systems Research, University of Birmingham, Birmingham, United Kingdom; 2 Centre for Endocrinology, Diabetes and Metabolism, Birmingham Health Partners, Birmingham, United Kingdom; 3 Department of Endocrinology, Queen Elizabeth Hospital Birmingham, Birmingham, United Kingdom; 4 School of Mathematics, University of Birmingham, Birmingham, United Kingdom; 5 Department of Nuclear Medicine, Queen Elizabeth Hospital Birmingham, Birmingham, United Kingdom; 6 Department of Clinical Biochemistry, University Hospital South Manchester NHS Foundation Trust, Manchester Academic Health Science Centre, University of Manchester, Manchester, United Kingdom; 7 Faculty of Medicine and Health, Worsley Building, University of Leeds, Leeds, United Kingdom; Roswell Park Cancer Institute, UNITED STATES

## Abstract

Age-associated decline in muscle function represents a significant public health burden. Vitamin D-deficiency is also prevalent in aging subjects, and has been linked to loss of muscle mass and strength (sarcopenia), but the precise role of specific vitamin D metabolites in determining muscle phenotype and function is still unclear. To address this we quantified serum concentrations of multiple vitamin D metabolites, and assessed the impact of these metabolites on body composition/muscle function parameters, and muscle biopsy gene expression in a retrospective study of a cohort of healthy volunteers. Active serum 1,25-dihydroxyvitamin D3 (1α,25(OH)_2_D3), but not inactive 25-hydroxyvitamin D3 (25OHD3), correlated positively with measures of lower limb strength including power (rho = 0.42, p = 0.02), velocity (Vmax, rho = 0.40, p = 0.02) and jump height (rho = 0.36, p = 0.04). Lean mass correlated positively with 1α,25(OH)_2_D3 (rho = 0.47, p = 0.02), in women. Serum 25OHD3 and inactive 24,25-dihydroxyvitamin D3 (24,25(OH)_2_D3) had an inverse relationship with body fat (rho = -0.30, p = 0.02 and rho = -0.33, p = 0.01, respectively). Serum 25OHD3 and 24,25(OH)_2_D3 were also correlated with urinary steroid metabolites, suggesting a link with glucocorticoid metabolism. PCR array analysis of 92 muscle genes identified vitamin D receptor (*VDR*) mRNA in all muscle biopsies, with this expression being negatively correlated with serum 25OHD3, and Vmax, and positively correlated with fat mass. Of the other 91 muscle genes analysed by PCR array, 24 were positively correlated with 25OHD3, but only 4 were correlated with active 1α,25(OH)_2_D3. These data show that although 25OHD3 has potent actions on muscle gene expression, the circulating concentrations of this metabolite are more closely linked to body fat mass, suggesting that 25OHD3 can influence muscle function via indirect effects on adipose tissue. By contrast, serum 1α,25(OH)_2_D3 has limited effects on muscle gene expression, but is associated with increased muscle strength and lean mass in women. These pleiotropic effects of the vitamin D ‘metabolome’ on muscle function indicate that future supplementation studies should not be restricted to conventional analysis of the major circulating form of vitamin D, 25OHD3.

## Introduction

The effects of vitamin D on calcium homeostasis and bone health are well established. In recent years there has been great interest in its non-skeletal actions, with growing evidence from epidemiological, basic and clinical studies that vitamin D status is associated with effects including those on muscle function, body fat, immunity and cardiovascular disease risk [1]. Myopathy has long-been recognised to co-exist with reduced bone mineralization in the severe vitamin D deficiency states of rickets and osteomalacia [2]. In view of the great public health burden of so-called ‘sarcopenia’ and age-associated declines in muscle strength and function, there is significant interest in whether vitamin D may have a role in improving the healthy lifespan. Recent meta-analyses indicate that vitamin D supplementation in deficient elderly individuals reduces risk of falls [3]. There is also some evidence of beneficial effects on muscle strength and physical performance, however this is limited by heterogeneity of study designs, so that current guidelines do not recommend vitamin D supplementation for this indication [4–6].

Although basic research using cell culture and animal models has identified pathways by which vitamin D impacts upon muscle function, the situation in humans requires further delineation. In particular, although biopsy studies have demonstrated changes in muscle morphology in vitamin D deficient disease states, detailed analyses of the relationship between vitamin D status and gene expression of muscle atrophy markers are lacking [1]. There is also debate as to the optimal circulating levels of vitamin D, with further data on the impacts on human health and function required [5]. Furthermore in clinical practice, vitamin D status is defined by measurement of a single metabolite, 25-hydroxyvitamin D3 (25OHD3). Recently developed high-throughput liquid chromatography-tandem mass spectrometry (LC-MS/MS) techniques allow the quantification of multiple vitamin D metabolites, and to date this approach has not been used to assess their relationship with markers of muscle mass and function [7]. With these observations in mind, the aim of the current study was to perform an in-depth analysis of the relationship between serum vitamin D metabolites and muscle phenotype in a healthy human cohort.

## Materials and methods

### Subjects

116 Healthy human volunteers (79 women, 37 men; aged 20–74 years) recruited from local populations underwent a study protocol as outlined previously and had vitamin D analysis performed [8]. Briefly, significant medical conditions such as diabetes mellitus, vascular disease, epilepsy, malignancy and inflammatory diseases, use of oral anticoagulants and pregnancy, were exclusion criteria. Subjects arrived at the National Institute for Health Research (NIHR)-Wellcome Trust Clinical Research Facility at the Queen Elizabeth Hospital Birmingham, UK in a fasted state and completed a 1-day study protocol. Baseline observations were carried out and venous blood was obtained at the start of the study visit.

### Serum high-throughput vitamin D metabolite analysis by liquid chromatography tandem-mass spectrometry

Measurement of vitamin D metabolites was performed by liquid chromatography tandem-mass spectrometry (LC-MS/MS) as previously described [7]. Briefly, samples were prepared for analysis by protein precipitation followed by supportive liquid-liquid extraction (SLE). LC-MS/MS analysis was performed on a Waters AQUITY UPLC coupled to a Xevo TQ-S mass spectrometer. A Lux Cellulose-3 chiral column (100 mm, 2 mm, 3 μm) was used for separation, heated at 60°C in a column oven. The mobile phase was methanol/water/0.1% formic acid solution at a flow rate of 330 μl/min. The method was validated in accordance to US Food and Drug Administration (FDA) guidelines for accuracy, precision, extraction recovery and matrix effects, lower limit of detection and quantitation [9]. The vitamin D metabolites quantified for this study included 25OHD3, 25OHD2, 3-epi-25OHD3, 1α,25(OH)_2_D3 and 24,25(OH)_2_D3.

### Urine steroid profiling by gas chromatography/mass spectrometry

Subjects provided a 24 hour urine collection prior to the study day and on receipt a 30 ml aliquot was transferred to a universal container and stored at -80°C pending analysis. Analysis by gas chromatography/mass spectrometry (GC/MS) was performed, as described previously [8].

### Dual-energy x-ray absorptiometry scan

Body composition analysis was performed using dual-energy x-ray absorptiometry (DXA) (Hologic Discovery with DXA software version Apex 3.0; Hologic Inc) (coefficient of variation for fat and lean mass, <3%).

### Muscle strength testing

*Hand-held dynamometry*: Peak absolute strength (kilograms) and relative handgrip strength (kilograms of force per kilogram of body weight) were measured in triplicate bilaterally using a dynamometer (Takei Instruments). *Jump-plate mechanography*: Measures of lower limb strength were obtained using a ground force reaction platform (Leonardo System, Novotec) under the supervision of a trained operator using a standard operating procedure [8].

### Vastus lateralis muscle biopsy

Biopsies (n = 85; 45 women and 40 men) were performed by a single investigator (Z.K.H-S.), using a percutaneous Bergstrom technique as described previously. Samples were snap frozen in liquid nitrogen immediately following sampling and stored at -80°C pending analysis.

### Quantitative (real-time) PCR array analyses

Applied Biosystems reagents and pre-made gene expression assays were used. 18s rRNA was used as a reference gene for singleplex analysis. Targets genes were FAM labelled, with the reference gene being VIC-labelled. Reactions were performed using the Biomark system on a dynamic array integrated fluidic circuit (Fluidigm, San Francisco CA) according to the manufacturer's protocols. Gene targets and assay IDs were as follows: *VDR* (Hs00172113_m1), *CYP27B1* Hs01096154_m1, *CYP24A1* Hs00167999_m1, *mTOR* (Hs00234508_m1), *MAFbx/Atrogin-1* (Hs01041408_m1), *p300* (Hs00914223_m1), *MuRF1* (Hs00261590_m1), *Calpain-1* (Hs00559804_m1), *Calpain-2* (Hs00965097_m1), *USP19* (Hs00324123_m1), *ATF4* (Hs00909569_g1), *Caspase 3* (Hs00234387_m1), *eIF4BP1* (Hs00607050_m1), *FOXO1* (Hs01054576_m1), *FOXO3* (Hs00818121_m1), *MYH1* (Hs00428600_m1), *MYH2* (Hs00430042_m1), *MYH4* (Hs00757977_m1), *myogenin* (Hs01072232_m1), *SIRT1* (Hs01009005_m1), *SIRT3* (Hs00202030_m1), *myostatin* (Hs00976237_m1), *SMAD2* (Hs00183425_m1), *SMAD3* (Hs00969210_m1), *SMAD4* (Hs00929647_m1), *SMAD7* (Hs00998193_m1), *ACVR2A* (Hs00155658_m1), *ACVR2B* (Hs00609603_m1), *RELA* (Hs00153294_m1), *RELB* (Hs00232399_m1), *IL6* (Hs00985639_m1), *IL1B* (Hs01555410_m1), *NFKB1* (Hs00765730_m1), *INSR* (Hs00961554_m1), *IRS1* (Hs00178563_m1), *AKT1* (Hs00178289_m1), *GSK3B* (Hs01047719_m1), *DDIT4* (Hs01111686_g1), *HSD11B1* (Hs01547870_m1), *HSD11B2* (Hs00388669_m1), *H6PD* (Hs00188728_m1), *HSP90AA1* (Hs00743767_sH), *HSP90B1* (Hs00427665_g1), *GHR* (Hs00174872_m1), *GHSR* (Hs00269780_s1), *IGF1* (Hs01547656_m1), *HIF1A* (Hs00153153_m1), *EIF6* (Hs00158272_m1), *EIF2B* (Hs00426752_m1), *PDK4* (Hs01037712_m1), *IGF1R* (Hs00609566_m1), *GADD45A* (Hs00169255_m1), *ACACA* (Hs01046047), *AR* (Hs00171172_m1), *CD36* (Hs00169627_m1), *CDNK1* (Hs00355782_m1), *CEBP1* (Hs00270923_s1), *CRYAB* (Hs00157107_m1), *CYSC* (Hs01588974_g1), *GLUL* (Hs00365928_g1), *HSL* (Hs00193510_m1), *LPL* (Hs00173425_m1), *MYCL1* (Hs00420495_m1), *MYF5* (Hs00929416_g1), *NRC31* (Hs00354508_m1), *PPARD* (Hs04187066_g1), *PPARG* (Hs00234592_m1), *PPARGC1A* (Hs01016719_m1), *PPP3R2* (Hs00931245_s1), *PSMA2* (Hs00746751_s1), *PSMC1* (Hs02386942_g1), *PSMC2* (Hs00739800_m1), *PSMC4* (Hs00197826_m1), *PSMC5* (Hs01029472_g1), *PSMC6* (Hs01652481_g1), *PSMD1* (Hs00160631_m1), *PSMD2* (Hs01092076_g1), *PSMD3* (Hs00160646_m1), *PSMD4* (Hs01937833_s1), *PSMD6* (Hs00207850_m1), *PSMD7* (Hs00427396_m1), *PSMD11* (Hs00160660_m1), *PSMD12* (Hs00356667_m1), *PSMD14* (Hs01113429_m1), *RPS6KB1* (Hs00177357_m1), *RXRG* (Hs00199455_m1), *SLC2A4* (Hs00168966_m1), *SOCSS3* (Hs00168966_m1), *SOD1* (Hs00533490_m1), *SREBF1* (Hs01088691_m1), *TGFB1* (Hs00998133_m1), *TNFA* (Hs01113624_g1), *TRIM54* (Hs00936695_m1).

### Statistical analysis

Analyses were performed using Prism for Mac version 5.0 (GraphPad Software Inc) unless otherwise stated. For PCR analyses, statistical tests were performed on ΔCT values. Data were expressed in arbitrary units calculated by the formula 1000 × (2^−ΔCT^), or fold-change vs the 20- to 40-year age group (2^−ΔΔCT^). Nonparametric tests were used with Kruskal-Wallis and Dunn's post-test correction when comparing multiple groups. Bivariate correlations between variables were performed using Spearman's test. The LC/MS-MS lower detection limit of 32 pg/ml resulted in left-censored data for the 1α,25(OH)_2_D3 metabolite with 42 samples above the lower limit of detection. Further correlation analysis between this metabolite and gene expression levels was carried out using the statistical package *R* [10] and the function *cenken* from the *NADA* package [11]. Multivariate visualisation of correlations between metabolites of vitamin D were carried out with the R function scatterplot matrix from the *car* package [12], with values <32 pg/ml plotted as 32; trend-lines were computed using built-in robust fitting. Multiple linear regression analysis of performance measures on age, gender, BMI, and the metabolites 25OHD3 and 1α,25(OH)_2_D3 was carried out with the R package *leaps* [13], with 1α,25(OH)_2_D3 coded as 0 below detection limit of 32 pg/ml and 1 above detection limit. The best models with 1–5 variables included were constructed and variables with largest adjusted R^2^ values reported. Confidence limits for the Spearman rank correlation coefficient were calculated using GraphPad Prism, based on Fisher’s transformation [14].

### Ethical approval

The Coventry and Warwickshire Research Ethics Committee (REC reference no. 07/H1211/168) and the Scientific Committee of the NIHR-Wellcome Trust Clinical Research Facility at Queen Elizabeth Hospital Birmingham, UK approved the study functioning according to the guidelines on the Practice of Ethical Committees in Medical Research issued by the Royal College of Physicians of London. Recruitment ran from October 2010 to March 2013. Volunteers were provided with written and verbal information and gave written informed consent. After study completion, they received travel expenses, and clinically relevant results were communicated to general practitioners.

## Results

### Subject characteristics

**Fig 1:**
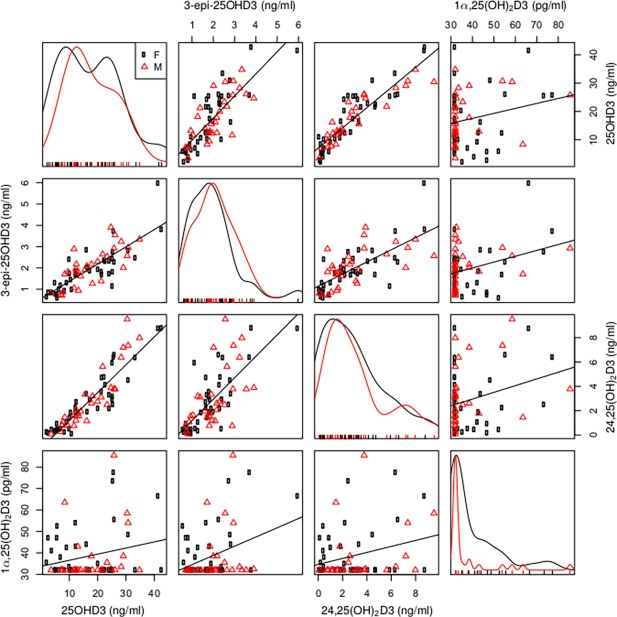
Multivariate visualisation of correlations between serum vitamin D metabolites. Serum 25OHD3 (ng/ml), 3-Epi-25OHD3 (ng/ml), 24,25(OH)_2_D3 (ng/ml) and 1 α,25(OH)_2_D3 (pg/ml) represented as a scatterplot matrix (black circles female, red triangles male), with robust linear trendlines for bivariate regressions. The curve plots on the main diagonal are univariate histograms; the off-diagonal panels are bivariate scatterplots between variables labelled at the plot edges (e.g. the top row, second-from-right panel is 24,25(OH)_2_D3 versus 25OHD3).

Subjects were spread across a broad age range with men and women represented (Table 1). The cohort was healthy and non-obese, with blood pressure and body composition profiles reflecting this. On grip strength measures as per European guidelines [15] only 4 females and none of the men met criteria for sarcopenia. Serum analysis of multiple vitamin D metabolites, revealed that 58% of the cohort were vitamin D deficient (total serum 25OHD <20 ng/ml, taking into account 25OHD2, 3-epi-25OHD3 and 25OHD3), 28% had insufficiency (20–30 ng/ml) and 14% had normal levels (> 30ng/ml) on Endocrine Society guideline criteria. Whilst 42% had normal levels based on Institute of Medicine criteria (>20ng/ml) [5,16]. Serum concentrations of 1α,25(OH)_2_D3 were quantifiable (> 32 pg/ml) in 38 subjects. Comparative analysis of serum vitamin D metabolites showed statistically significant correlations between 25OHD3, 24,25(OH)_2_D3 and 3-epi-25OHD3 (rho = 0.92, p<0.0001 and rho = 0.72, p<0.0001 respectively); and between 24,25(OH)_2_D3 and 3-epi-25OHD3 (rho = 0.62, p<0.0001) (Fig 1). There was no significant correlation between serum 1α,25(OH)_2_D3 and concentrations of other vitamin D metabolites (Fig 1).

**Table 1:** Subject characteristics for overall group. Data are expressed as median (IQR). SBP = systolic blood pressure, DBP = diastolic blood pressure, BMI = body mass index.

	Median	IQR
**Demographics**		
Age (years)	44.0	27.3–60.0
**Observational Data**		
SBP (mmHg)	127.0	116.5–140.5
DBP (mmHg)	77.0	68.0–87.0
BMI (kg/m^2^)	24.7	22.5–27.1
**Body Composition**		
Total Fat Mass (kg)	18.8	15.2–23.4
Total Lean Mass (kg)	45.0	40.2–57.6
Bone Mass (BMC) (kg)	2.2	2.0–2.5
Body Fat (%)	28.2	22.4–34.5
**Serum Vitamin D Metabolite**		
25OHD3 (ng/ml)	14.0	8.6–23.0
1α,25(OH)_2_D3 (pg/ml)	44.0	37.0–54.0
24,25(OH)_2_D3 (ng/ml)	2.0	0.8–3.8
3-Epi-25OHD3 (ng/ml)	1.8	1.1–2.3
25OHD2 (ng/ml)	0.7	0.4–2.0
23,25(OH)_2_D3 (ng/ml)	0.3	0.2–0.5

### Vitamin D metabolites and body composition and biochemical parameters

**Fig 2:**
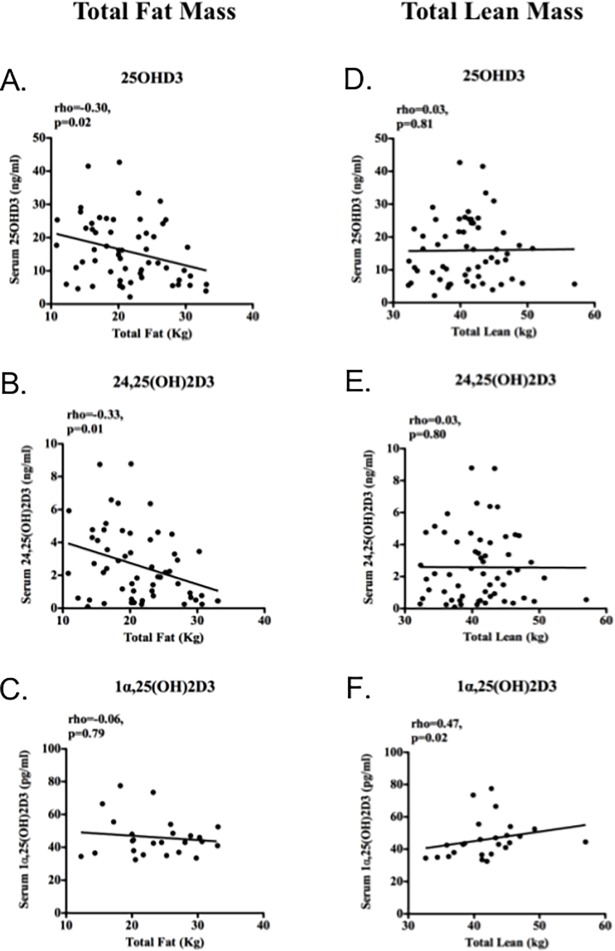
Serum vitamin D metabolites and body composition parameters in women. Serum (a) 25OHD3, (b) 24,25(OH)_2_D3 correlated negatively with body fat (rho = -0.30, p = 0.02 and rho = -0.33, p = 0.01, respectively), but not (c) 1α,25(OH)_2_D3 (rho = -0.06, p = 0.79). Conversely, lean mass correlated positively with (f) 1α,25(OH)_2_D3 (rho = 0.47, p = 0.02), but not (d) 25OHD3 or (e) 24,25(OH)_2_D3 (rho = 0.03, p = 0.81, and rho = 0.03, p = 0.80). Data were analysed by Spearman correlations (rho) with p values and line of best fit shown.

In the group as a whole, there was a significant negative correlation between serum 25OHD3 and body fat (%) (rho = -0.20, 95%CI -0.40–0.00, p = 0.04). In women, total fat mass was negatively correlated with serum 25OHD3 and 24,25(OH)_2_D3, but not 1α,25(OH)_2_D3 (Fig 2A–2C). Conversely 1α,25(OH)_2_D3 was positively correlated with total lean mass, whilst the other metabolites were not (Fig 2D–2F). In men, no significant associations were observed between vitamin D metabolites and body composition parameters (serum 25OHD3 vs. total fat mass rho = 0.01, 95% CI -0.33–0.35, p = 0.94, and vs. lean mass rho = 0.09, 95% CI -0.25–0.42, p = 0.60; serum 1α,25(OH)_2_D3 vs. total fat mass rho = -0.36, 95% CI -0.78–0.29, p = 0.26, vs. lean mass rho = 0.20, 95% CI -0.16–0.50, p = 0.26). In women, 25OHD3 was also negatively correlated with BMI (rho = -0.34, 95% CI -0.58–-0.04, p = 0.02) and positively correlated with HDL-C (rho = 0.33, 95% CI 0.06–0.55, p = 0.01).

**Fig 3:**
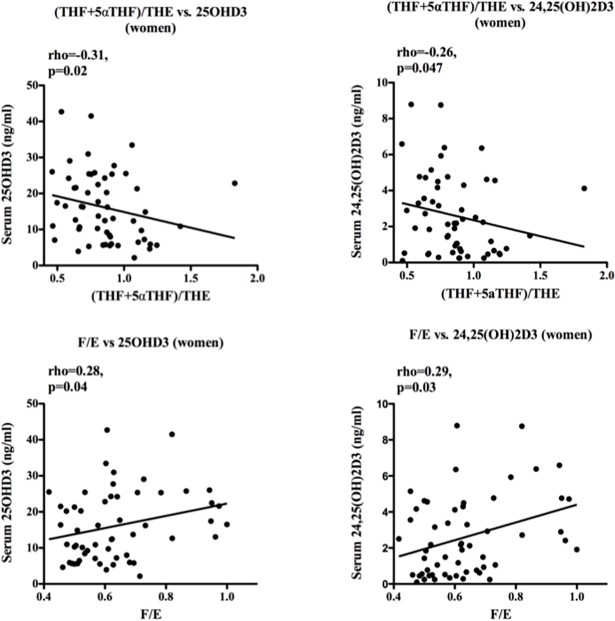
Serum vitamin D metabolites and urinary steroid metabolism in women. Serum 25OHD3 and 24,25(OH)_2_D3 correlated negatively with urinary (tetrahydrocortisol+5α tetrahydrocortisol)/ tetrahydrocortisone ((THF+5αTHF)/THE ratios, and positively with urinary cortisol/cortisone (F/E) ratios. Data were analysed by Spearman correlations (rho) with p values and line of best fit shown.

Analysis of urinary steroid metabolites showed a significant negative correlation between serum 25OHD3 and 24,25(OH)_2_D3 and (tetrahydrocortisol+5α tetrahydrocortisol)/ tetrahydrocortisone ((THF+5αTHF)/THE ratios (rho = -0.31, p = 0.02, and rho = -0.26, p = 0.047 respectively), and positive correlation with cortisol/cortisone (F/E) (rho = 0.28, p = 0.04, and rho = 0.29, p = 0.03 respectively), indicative of negative associations with 11β-hydroxysteroid dehydrogenase (11β-HSD) type 1 and type 2 activities, respectively (Fig 3). These associations were not seen in men, and not observed for 1,25(OH)_2_D3 (data not shown). No significant relationships between serum 25OHD3 or 1α,25(OH)_2_D3 and fasting glucose, insulin or HOMA-IR were observed.

### Vitamin D metabolites and muscle function parameters

**Fig 4:**
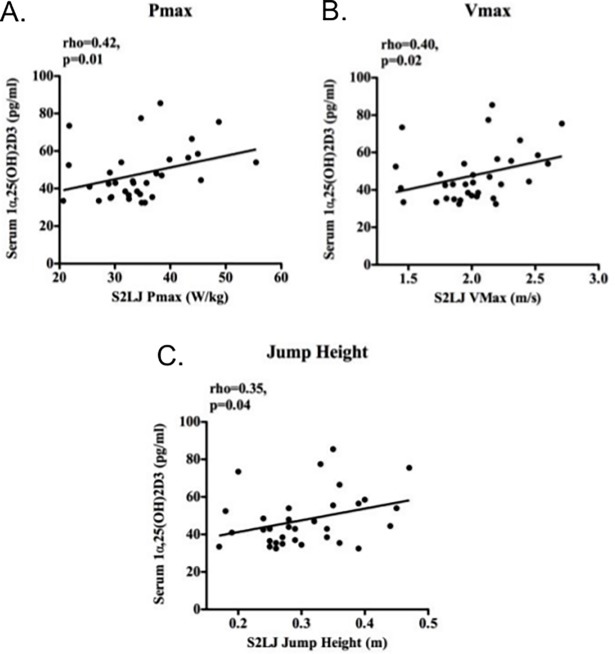
Serum active vitamin D and muscle strength. 1α,25(OH)_2_D3 correlated positively with jump plate measures of lower limb strength (a) Pmax (maximal power), (b) Vmax (maximum velocity), (c) jump height, all on standing 2-legged jump (S2LJ). Data were analysed by Spearman correlations (rho) with line of best fit shown.

In contrast to precursor 25OHD3, serum 1α,25(OH)_2_D3 concentrations correlated positively with jump-plate mechanography measures including power (Pmax), velocity (Vmax) and jump height (Fig 4A, 4B and 4C respectively and Table 2). Conversely, whereas 1α,25(OH)_2_D3 had no significant effect on the efficiency or ‘Esslinger’ performance index, which gives a measure of performance controlled for body weight, 25OHD3 and 24,25(OH)_2_D3 were positively correlated with these measures (Table 2).

**Table 2:** Bivariate correlations between serum vitamin D metabolites and subject characteristics.

	Correlation Coefficients (Spearman)						
		1α,25(OH)_2_D3	25OHD3	24,25(OH)_2_D3	3-Epi-25OHD3	25OHD2	23,25(OH)_2_D3
**Strength testing**							
Pmax		0.42* **(0.08–0.67, p = 0.02)**	0.03 (-0.21–0.26, p = 0.81)	0.10 (-0.14–0.32, p = 0.42)	0.04 (-0.19–0.28, p = 0.68)	-0.24 (-0.53–0.09, p = 0.14)	0.06 (-0.38–0.47, p = 0.80)
Vmax		0.40* **(0.06–0.66, p = 0.02)**	0.07 (-0.17–0.30, p = 0.56)	0.11 (-0.13–0.34, p = 0.34)	0.09 (-0.15–0.32, p = 0.47)	-0.17 (-0.47–0.16, p = 0.29)	0.16 (-0.28–0.55, p = 0.46)
Jump Height		0.36* **(0.00–0.63, p = 0.04)**	0.02 (-0.21–0.25, p = 0.84)	0.06 (-0.18–0.29, p = 0.61)	0.03 (-0.21–0.27, p = 0.79)	-0.11 (-0.42–0.22, p = 0.51)	0.13 (-0.31–0.53, p = 0.54)
Efficiency		0.18 (-0.18–0.50, p = 0.31)	0.36** **(0.13–0.55, p = 0.002)**	0.34** **(0.11–0.53, p = 0.003)**	0.21 (-0.02–0.43, p = 0.07)	0.10 (-0.24–0.41, p = 0.56)	0.01 (-0.41–0.43, p = 0.96)
Esslinger Index		0.30 (-0.06–0.58, p = 0.09)	0.25* **(0.01–0.46, p = 0.03)**	0.29* **(0.06–0.49, p = 0.01)**	0.12 (-0.12–0.35, p = 0.30)	0.01 (-0.32–0.33, p = 0.97)	-0.15 (-0.54–0.28, p = 0.48)

Data are Spearman correlation coefficients (rho)), with 95% confidence intervals (CI) and *p* values in brackets

*p<0.05

**p<0.01).

No significant correlations between other vitamin D metabolites and strength testing measures were observed. Multiple linear regression analysis revealed that age (jump-height p = 2.45e-8, Vmax p = 1.35e-7) and gender (jump-height p = 8.73e-9, Vmax p = 1.10e-9 and grip strength p = <2e-16) were the strongest predictors of strength testing measures and that these findings were highly significant. Furthermore, 1α,25(OH)_2_D3 coded as below/above detection limit provided an improved model for jump height when added to age and gender, assessed by adjusted R^2^ value, but fell short of statistical significance for the data available alone (p = 0.128).

### 25OHD3, 1,25(OH)_2_D3, VDR and skeletal muscle gene expression

**Fig 5:**
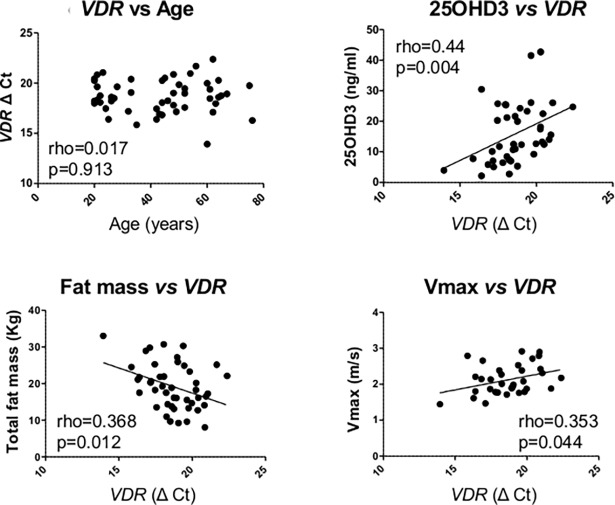
Expression of *VDR* mRNA in human muscle biopsies. Relationship between muscle expression of VDR (RT-PCR ΔCt value) and: age; serum 25OHD3 concentrations (ng/ml); fat mass (Total mass, kg); Vmax (m/s). p values for linear regression analyses are shown and significant correlations are shown as solid lines. Data were analysed by Spearman correlations (rho) with line of best fit shown.

RT-PCR analysis of skeletal muscle mRNA showed that there was no detectable expression of *CYP27B1*, the gene encoding the enzyme 1α-hydroxylase that catalyzes conversion of 25OHD3 to 1α,25(OH)_2_D3 (data not shown). In a similar fashion, mRNA for the vitamin D catabolic enzyme 24-hydroxylase (*CYP24A1*) was also undetectable in the muscle biopsies (data not shown). However, mRNA for the nuclear receptor for 1α,25(OH)_2_D3 (vitamin D receptor, *VDR*) was detectable in the muscle biopsies (mean ΔCT = 18.7, 17.6–19.9), and correlated with specific muscle and fat parameters. Muscle *VDR* expression was not significantly affected by donor age, but correlated negatively with serum 25OHD3 concentrations and muscle Vmax measurements, and positively with fat mass (Fig 5). There was no significant correlation between *VDR* mRNA expression and serum 1α,25(OH)_2_D3, lean mass, BMC, jump height, grip strength or Vmax (data not shown). However, *VDR* mRNA expression was positively correlated with 21 of the 91 other gene targets analysed by PCR array (Table 3).

**Table 3:** Bivariate correlations between expression of Vitamin D Receptor (*VDR*) and other skeletal muscle target genes. Data are Spearman correlation coefficients (rho)), with 95% confidence intervals (CI) and *p* values in brackets).

	VDR Expression		
Gene	rho	95% CI	p-value
*mTOR*	-0.18	-0.44–0.10	0.20
*Atrogin1*	-0.24	-0.49–0.04	0.08
*P300*	0.24	-0.04–0.49	0.08
*MuRF1*	-0.23	-0.48–0.05	0.10
*Calpain1*	-0.26	-0.51–0.02	0.06
*Calpain2*	-0.18	-0.44–0.11	0.21
*USP19*	-0.27	-0.51–0.01	0.05
*ATF-4*	-0.15	-0.42–0.14	0.28
*Caspase3*	0.15	-0.14–0.42	0.28
*eIF4BP1*	-0.23	-0.49–0.05	0.10
*FOXO1*	-0.22	-0.48–0.06	0.12
*FOXO3*	-0.20	-0.45–0.09	0.16
*MYH1*	-0.24	-0.49–0.04	0.09
***MYH2***	**-0.36**	**-0.58–-0.08**	**0.009**
*MYH4*	-0.21	-0.47–0.08	0.13
*Myogenin*	-0.06	-0.33–0.23	0.69
*SIRT1*	-0.22	-0.47–0.07	0.12
***SIRT3***	**-0.30**	**-0.54–-0.02**	**0.03**
***Myostatin***	**-0.32**	**-0.55–-0.04**	**0.02**
***SMAD2***	**0.32**	**0.04–0.55**	**0.02**
*SMAD3*	-0.25	-0.50–0.03	0.07
***SMAD4***	**-0.33**	**-0.56–-0.05**	**0.02**
***SMAD7***	**0.33**	**0.06–0.56**	**0.02**
***ACVR2A***	**-0.33**	**-0.56–-0.05**	**0.02**
*ACVR2B*	-0.25	-0.50–0.03	0.07
*RELA*	-0.22	-0.47–0.06	0.11
*RELB*	-0.01	-0.29–0.27	0.95
*IL6*	0.07	-0.21–0.35	0.59
*IL1B*	0.17	-0.12–0.43	0.24
*NFKB1*	-0.21	-0.46–0.08	0.15
*INSR*	-0.23	-0.48–0.05	0.11
***IRS1***	**-0.34**	**-0.57–-0.07**	**0.01**
*AKT1*	-0.18	-0.44–0.11	0.21
*GSK3B*	-0.18	-0.44–0.11	0.21
***DDIT4***	**-0.36**	**-0.59–-0.09**	**0.009**
*HSD11B1*	-0.15	-0.41–0.14	0.30
*HSD11B2*	-0.06	-0.34–0.22	0.68
*H6PD*	-0.27	-0.51–0.02	0.06
*HSP90AA1*	-0.21	-0.46–0.08	0.14
*HSP90B1*	-0.05	-0.33–0.23	0.74
***GHR***	**-0.30**	**-0.54–-0.02**	**0.03**
*GHSR*	0.25	-0.04–0.50	0.08
*IGF1*	0.02	-0.27–0.30	0.90
*HIF1A*	-0.04	-0.32–0.25	0.79
*EIF6*	-0.11	-0.39–0.17	0.42
*EIF2B1*	-0.21	-0.47–0.07	0.14
*PDK4*	-0.10	-0.37–0.19	0.50
*IGF1R*	-0.25	-0.49–0.04	0.08
*GADD45A*	-0.15	-0.41–0.14	0.30
*ACACA*	-0.17	-0.44–0.12	0.22
*AR*	-0.23	-0.48–0.06	0.11
*CD36*	-0.04	-0.32–0.25	0.80
*CDKN1A*	0.15	-0.13–0.42	0.28
***CEBPB***	**-0.31**	**-0.55–-0.03**	**0.03**
*CRYAB*	-0.14	-0.41–0.15	0.33
***CYCS***	**-0.31**	**-0.55–-0.03**	**0.02**
***GLUL***	**-0.36**	**-0.58–-0.08**	**0.01**
*HSL*	-0.19	-0.45–0.10	0.18
*LPL*	0.03	-0.26–0.31	0.86
*MYCL1*	-0.18	-0.44–0.11	0.21
*MYF5*	-0.13	-0.40–0.16	0.35
*NR3C1*	0.17	-0.12–0.42	0.25
*PPARD*	-0.21	-0.47–0.07	0.13
*PPARG*	0.01	-0.27–0.29	0.93
***PPARGC1A***	**-0.34**	**-0.57–-0.06**	**0.02**
*PPP3R2*	0.28	-0.00–0.51	0.05
*PSMA2*	-0.03	-0.31–0.25	0.86
*PSMC1*	-0.24	-0.49–0.05	0.09
*PSMC2*	-0.24	-0.49–0.05	0.09
*PSMC4*	-0.27	-0.52–0.01	0.05
***PSMC5***	**-0.34**	**-0.57–-0.07**	**0.01**
*PSMC6*	-0.20	-0.45–0.09	0.17
*PSMD1*	-0.26	-0.51–0.03	0.07
*PSMD11*	-0.27	-0.52–0.01	0.05
*PSMD12*	-0.24	-0.49–0.05	0.10
***PSMD14***	**-0.30**	**-0.54–-0.02**	**0.03**
***PSMD2***	**-0.29**	**-0.53–-0.01**	**0.04**
*PSMD3*	-0.20	-0.45–0.09	0.17
*PSMD4*	0.05	-0.24–0.33	0.74
*PSMD6*	-0.26	-0.50–0.03	0.07
*PSMD7*	-0.25	-0.50–0.03	0.07
***RPS6KB1***	**-0.31**	**-0.54–-0.03**	**0.03**
***RXRG***	**-0.30**	**-0.54–-0.01**	**0.03**
*SLC2A4*	-0.27	-0.51–0.02	0.06
*SOCSS3*	-0.08	-0.36–0.21	0.58
***SOD1***	**-0.30**	**-0.54–-0.01**	**0.03**
*SREBF1*	-0.22	-0.48–0.07	0.12
*TGFB1*	-0.13	-0.40–0.16	0.37
*TNFA*	-0.02	-0.30–0.26	0.90
***TRIM54***	**-0.28**	**-0.52–0.00**	**0.04**

Of the muscle genes analysed by PCR array 24 correlated positively with serum 25OHD3 (Table 4 and S1 Table). By contrast, only four genes correlated positively with serum concentrations of 1α,25(OH)_2_D3 (Table 4). When additional analyses to account for censored data were performed a total of eight genes correlated positively with 1α,25(OH)_2_D3: LPL(p = 0.005), CD36(p = 0.01), PPARG(p = 0.01), HSP90B1(p = 0.01), PPP3R2(p = 0.02), PDK4(p = 0.03), FOXO3(p = 0.03), FOXO1(p = 0.04). There was no overlap between genes that correlated with serum 25OHD3 and those that correlated with serum 1α,25(OH)_2_D3.

**Table 4:** Bivariate correlations between serum 25OHD3, 1α,25(OH)_2_D3 and gene expression in skeletal muscle. Data are Spearman correlation coefficients (rho)), with 95% confidence intervals (CI) and *p* values in brackets).

	Serum 25OHD3			Serum 1α,25(OH)_2_D3		
Gene	rho	95% CI	p-value	rho	95% CI	p-value
***VDR***	**-0.41**	**-0.64–-0.10**	**0.008**	-0.08	-0.58–0.47	0.78
*mTOR*	0.06	-0.22–0.33	0.68	0.22	-0.26–0.61	0.36
*Atrogin1*	0.08	-0.20–0.34	0.58	-0.24	-0.62–0.24	0.32
*P300*	-0.08	-0.35–0.20	0.58	0.24	-0.24–0.62	0.32
*MuRF1*	0.15	-0.13–0.41	0.28	-0.16	-0.57–0.32	0.50
*Calpain1*	0.16	-0.11–0.42	0.23	-0.03	-0.48–0.43	0.88
*Calpain2*	0.20	-0.08–0.45	0.14	-0.08	-0.52–0.39	0.73
*USP19*	0.27	-0.01–0.50	0.05	0.12	-0.36–0.54	0.63
*ATF-4*	0.22	-0.06–0.46	0.11	-0.17	-0.58–0.31	0.49
*Caspase3*	-0.22	-0.47–0.06	0.11	0.17	-0.31–0.58	0.49
***eIF4BP1***	**0.37**	**0.10–0.59**	**0.006**	0.15	-0.33–0.56	0.53
*FOXO1*	0.25	-0.03–0.49	0.07	-0.03	-0.48–0.43	0.91
*FOXO3*	0.26	-0.02–0.50	0.05	-0.05	-0.49–0.41	0.83
*MYH1*	0.27	-0.01–0.51	0.05	-0.02	-0.47–0.43	0.93
***MYH2***	**0.30**	**0.03–0.54**	**0.03**	0.01	-0.45–0.46	0.98
*MYH4*	0.12	-0.16–0.39	0.38	-0.17	-0.58–0.31	0.48
***Myogenin***	**0.27**	**-0.00–0.51**	**0.047**	0.10	-0.37–0.53	0.68
*SIRT1*	0.22	-0.07–0.46	0.12	-0.35	-0.69–0.12	0.12
*SIRT3*	0.16	-0.13–0.41	0.26	-0.08	-0.52–0.38	0.73
*Myostatin*	0.13	-0.16–0.39	0.36	-0.22	-0.61–0.26	0.36
*SMAD2*	-0.13	-0.39–0.16	0.36	0.22	-0.26–0.61	0.36
***SMAD3***	**0.32**	**0.04–0.54**	**0.02**	-0.21	-0.61–0.26	0.37
***SMAD4***	**0.28**	**-0.00–0.51**	**0.04**	-0.10	-0.53–0.37	0.67
***SMAD7***	**-0.28**	**-0.51–0.00**	**0.04**	0.10	-0.37–0.52	0.67
***ACVR2A***	**0.27**	**-0.00–0.51**	**0.046**	-0.18	-0.58–0.29	0.45
*ACVR2B*	0.26	-0.02–0.50	0.06	-0.12	-0.54–0.35	0.62
*RELA*	0.15	-0.13–0.40	0.29	-0.15	-0.56–0.33	0.54
*RELB*	0.08	-0.21–0.34	0.60	0.08	-0.39–0.51	0.74
*IL6*	-0.26	-0.49–0.02	0.06	0.29	-0.18–0.66	0.21
*IL1B*	-0.16	-0.42–0.12	0.24	-0.02	-0.47–0.43	0.93
*NFKB1*	0.17	-0.12–0.42	0.23	-0.25	-0.63–0.23	0.29
*INSR*	0.31	0.03–0.54	0.02	0.11	-0.35–0.54	0.61
*IRS1*	0.20	-0.08–0.45	0.14	0.02	-0.44–0.47	0.94
*AKT1*	-0.09	-0.35–0.19	0.54	0.07	-0.40–0.50	0.77
*GSK3B*	0.20	-0.08–0.46	0.15	-0.19	-0.59–0.29	0.43
***DDIT4***	**0.31**	**0.04–0.55**	**0.02**	-0.09	-0.52–0.38	0.71
*HSD11B1*	0.15	-0.13–0.41	0.29	-0.40	-0.72–0.06	0.08
*HSD11B2*	-0.03	-0.31–0.25	0.83	-0.15	-0.57–0.32	0.53
*H6PD*	0.25	-0.03–0.49	0.07	-0.14	-0.56–0.33	0.55
***HSP90AA1***	**0.35**	**0.08–0.57**	**0.01**	0.21	-0.27–0.61	0.37
*HSP90B1*	0.12	-0.16–0.39	0.38	-0.14	-0.56–0.33	0.56
*GHR*	0.19	-0.09–0.44	0.17	-0.23	-0.62–0.24	0.33
*GHSR*	-0.12	-0.39–0.16	0.39	-0.35	-0.69–0.12	0.14
*IGF1*	-0.11	-0.38–0.17	0.42	-0.37	-0.70–0.10	0.11
*HIF1A*	0.16	-0.12–0.42	0.24	-0.26	-0.64–0.21	0.25
*EIF6*	-0.01	-0.29–0.27	0.94	0.06	-0.40–0.50	0.80
***EIF2B1***	**0.28**	**0.00–0.52**	**0.04**	-0.22	-0.61–0.26	0.35
*PDK4*	0.15	-0.13–0.41	0.27	0.31	-0.17–0.66	0.19
***IGF1R***	**0.35**	**0.08–0.58**	**0.009**	-0.14	-0.55–0.33	0.55
*GADD45A*	0.19	-0.10–0.44	0.18	-0.34	-0.69–0.13	0.15
***ACACA***	0.05	-0.22–0.32	0.71	**-0.48**	**-0.77–-0.03**	**0.03**
*AR*	0.22	-0.06–0.47	0.11	-0.20	-0.60–0.27	0.39
*CD36*	0.21	-0.07–0.46	0.12	0.12	-0.35–0.54	0.60
*CDKN1A*	-0.03	-0.30–0.25	0.82	-0.29	-0.66–0.19	0.22
*CEBPB*	0.26	-0.01–0.50	0.06	-0.16	-0.58–0.31	0.48
***CRYAB***	**0.34**	**0.07–0.56**	**0.01**	0.15	-0.32–0.56	0.52
***CYCS***	**0.39**	**0.13–0.60**	**0.004**	0.29	-0.18–0.66	0.21
***GLUL***	**0.35**	**0.08–0.57**	**0.01**	0.07	-0.39–0.51	0.75
*HSL*	0.08	-0.20–0.34	0.58	0.21	-0.27–0.60	0.38
*LPL*	0.15	-0.13–0.41	0.29	-0.01	-0.46–0.44	0.97
*MYCL1*	0.01	-0.26–0.28	0.93	0.01	-0.45–0.46	0.97
*MYF5*	0.21	-0.07–0.46	0.13	0.21	-0.27–0.60	0.38
*NR3C1*	-0.11	-0.37–0.17	0.43	-0.18	-0.59–0.30	0.45
***PPARD***	**0.28**	**0.00–0.52**	**0.04**	-0.17	-0.58–0.30	0.46
*PPARG*	-0.03	-0.30–0.25	0.82	-0.30	-0.66–0.18	0.20
***PPARGC1A***	**0.35**	**0.08–0.57**	**0.01**	0.11	-0.36–0.54	0.63
***PPP3R2***	-0.17	-0.42–0.11	0.23	**-0.53**	**-0.79–0.10**	**0.02**
***PSMA2***	0.09	-0.19–0.36	0.49	**-0.45**	**-0.75–0.06**	**0.04**
*PSMC1*	0.16	-0.12–0.42	0.24	-0.05	-0.50–0.40	0.82
*PSMC2*	0.24	-0.04–0.48	0.08	-0.18	-0.58–0.30	0.44
***PSMC4***	**0.27**	**0.00–0.51**	**0.048**	-0.09	-0.52–0.38	0.70
***PSMC5***	**0.28**	**0.00–0.51**	**0.04**	-0.05	-0.49–0.41	0.83
*PSMC6*	0.23	-0.05–0.47	0.10	0.08	-0.39–0.51	0.74
*PSMD1*	0.21	-0.07–0.46	0.12	-0.16	-0.57–0.32	0.51
***PSMD11***	**0.28**	**0.00–0.51**	**0.04**	-0.03	-0.48–0.42	0.88
***PSMD12***	**0.27**	**0.00–0.51**	**0.04**	0.01	-0.45–0.46	0.97
***PSMD14***	**0.33**	**0.06–0.55**	**0.02**	0.14	-0.34–0.56	0.56
*PSMD2*	0.18	-0.10–0.43	0.20	-0.08	-0.52–0.38	0.72
*PSMD3*	0.07	-0.21–0.34	0.60	-0.04	-0.48–0.42	0.87
*PSMD4*	0.04	-0.23–0.31	0.75	-0.37	-0.70–0.11	0.11
*PSMD6*	0.11	-0.17–0.37	0.42	0.14	-0.33–0.55	0.55
*PSMD7*	0.21	-0.07–0.45	0.13	-0.05	-0.49–0.41	0.82
*RPS6KB1*	0.24	-0.03–0.49	0.08	-0.18	-0.59–0.30	0.45
***RXRG***	**0.40**	**0.14–0.61**	**0.003**	0.25	-0.23–0.62	0.30
*SLC2A4*	0.19	-0.09–0.45	0.16	-0.05	-0.49–0.41	0.82
*SOCSS3*	-0.06	-0.33–0.22	0.65	0.10	-0.37–0.53	0.67
***SOD1***	**0.29**	**0.02–0.53**	**0.03**	-0.12	-0.55–0.35	0.60
***SREBF1***	0.08	-0.20–0.35	0.56	**-0.49**	**-0.77–-0.05**	**0.03**
*TGFB1*	-0.05	-0.32–0.22	0.70	-0.30	-0.66–0.18	0.21
*TNFA*	-0.09	-0.35–0.19	0.53	0.02	-0.43–0.47	0.92
*TRIM54*	0.23	-0.05–0.47	0.10	0.11	-0.37–0.53	0.65

## Discussion

To date the majority of cross-sectional studies of vitamin D and muscle function have focused on circulating concentrations of 25OHD3, with some reports describing positive correlations with a heterogenous range of muscle function measures and others describing no significant associations, as outlined by Girgis and colleagues [1]. However, it is important to recognise that 25OHD3 is a relatively inactive form of vitamin D, so that its effects on target tissues are dependent on either systemic or localised metabolism to other vitamin D metabolites. The aim of the current study was to clarify the metabolic mechanisms that underpin the actions of vitamin D in human muscle.

Use of 25OHD3 as the primary marker of ‘vitamin D status’ has obvious advantages in terms of longer half-life and serum stability, as well as the direct link between vitamin D supplementation/restriction and serum concentrations of 25OHD3. By contrast, serum concentrations of 1α,25(OH)_2_D3 are dependent not only on precursor 25OHD3, but also PTH, and fibroblast growth factor 23 (FGF23) that coordinate renal expression of the enzyme 25-hydroxyvitamin D-1α-hydroxylase (CYP27B1) [17]. This is underlined by data presented in the current study where there was no significant correlation between serum levels of 25OHD3 and 1α,25(OH)_2_D3. In contrast, serum 25OHD3, 24,25(OH)_2_D3 and 3-epi-25OHD3 levels were strongly correlated. Concentrations of 1α,25(OH)_2_D3 were not quantifiable in some serum samples, which reflects the overall low vitamin D status of the study cohort.

A small number of studies have assessed correlations between serum concentrations of 1α,25(OH)_2_D3, knee extension power [18] and reduced muscle mass and knee strength in females under the age of 65 years [19]. Other vitamin D metabolites such as 24,25(OH)_2_D3 and 3-epi-25OHD3 have not been previously studied in relation to muscle function. It was therefore interesting to note that circulating 1α,25(OH)_2_D3, was a better correlate of muscle strength than precursor 25OHD3. Conversely, serum 25OHD3 had a greater impact than 1α,25(OH)_2_D3 on other muscle markers such as efficiency (a measure of the relationship between maximum jump force and power, with the less force required to generate the same power, the more efficient the jump) and the Esslinger fitness index (Pmax relative to weight normalised to age and sex), as well as skeletal muscle gene expression. One potential explanation for this is that systemic and locally-generated 1α,25(OH)_2_D3 have different effects on muscle phenotype and function. It is interesting that both 25OHD3 and 24,25(OH)_2_D3 are associated with body fat in addition to efficiency and Esslinger fitness index. It may be that the added burden of fat in generating power and interaction with these metabolites plays a role in these relationships. In addition, as 1α,25(OH)_2_D3 is positively correlated with Pmax but does not reach significance with Esslinger, it is possible that age or sex impact these effects. It is also possible that observed sex differences are due to lack of statistical power in view of smaller numbers of men recruited to the study. The expression of *VDR* mRNA in muscle biopsies confirms previous observations for this receptor in human muscle and provides a mechanism for 1α,25(OH)_2_D3 responsiveness in muscle cells. Nevertheless, there is conflicting evidence as to the true levels of VDR protein expression in human skeletal muscle due to questions over antibody specificity [20,21]. Furthermore it has been suggested that VDR levels change across the life-course, but we found no evidence of this in our adult cohort.

Parallel analysis of the *CYP27B1* in muscle biopsies did not reveal significant expression of this gene (data not shown), suggesting that, unlike many other target tissues for vitamin D, localised conversion of 25OHD3 to 1α,25(OH)_2_D3 is not a prominent feature of muscle function. An alternative to this is that serum 25OHD3 exerts effects on muscle via an indirect mechanism(s). Previous work from our group has described the role of pre-receptor glucocorticoid metabolism via 11β-HSD1 in the muscle weakness of aging and steroid excess [8]. Our observation in the current study that increased serum 25OHD3, but not 1α,25(OH)_2_D3 was associated with reduced 11β-HSD1 activity highlights an important overlap between these two steroid hormone metabolic systems. Suppression of 11β-HSD1 activity by increased serum 25OHD3 may help to promote muscle function through decreased levels of sarcopenic cortisol. Although there is existing evidence for cross-talk between these systems in diverse contexts such as pregnancy, obesity, asthma and renal disease, no previous studies have focused on this in the context of muscle health and this represents an area of immediate interest for the development of mechanistic studies [22,23].

There is evidence of an inverse relationship between serum 25OHD3 and fat mass, such that weight loss in obesity is associated with increasing levels of 25OHD3. Volumetric dilution or sequestration of lipophilic vitamin D in adipose tissue are potential explanations for this effect, however the exact mechanisms regulating this are undefined [24]. We observed that 1α,25(OH)_2_D3 was associated with lean mass, and 25OHD3, and 24,25(OH)_2_D3 were associated with fat mass in women. The observation regarding lean mass is unsurprising in that this is closely associated with measures of muscle strength, which 1α,25(OH)_2_D3 is also correlated with. Low serum 25OHD3 has previously been associated with a metabolically unfavorable body composition, lipid and insulin resistance profile in post-menopausal women previously and investigation of causality and the effects of replacement are required [25].

This is the first published report of jump-plate mechanographic analysis of an adult cohort in relation to vitamin D. The advantages of this technique are that there is evidence of greater re-test/inter-rater reproducibility along with improved sensitivity for sarcopenia compared to traditional tests of muscle function [26]. Previous studies have used various isometric and physical performance measures to assess the impact of vitamin D on muscle function, with data focused primarily on serum concentrations of 25OHD3 [1,27]. It was therefore interesting in our healthy cohort, that active 1α,25(OH)_2_D3 (but not inactive 25OHD3) correlates with muscle markers of maximum power, velocity and jump height, which are influenced by the proportion of type II fast twitch muscle fibers. These fibers are preferentially lost in endocrine myopathies as exemplified by severe vitamin D deficiency [2,28]. In a previously reported adolescent female cohort jump-plate mechanography measures were shown to have a positive relationship with 25OHD [29]. Possible explanations for the absence of a significant relationship with grip strength observed in our cohort are that proximal lower limb muscle function is more sensitive to vitamin D status, as seen in the osteomalacia phenotype of which jump-plate measures would be more sensitive [15].

Early human studies described skeletal muscle morphological changes consisting of non-specific/type II fast-twitch fiber atrophy and degeneration, that were reversible with vitamin D supplementation in patients with proximal myopathy and osteomalacia [30]. However, literature on the relationship between vitamin D and human skeletal muscle gene expression changes is limited. We identified 24 skeletal muscle genes (out of 92 examined), which correlate with serum 25OHD3 (Table 4 and S1 Table). These genes are involved in hormonal/intracellular signalling, cell stress, proteosomal activity, protein translation, amino acid metabolism, muscle contraction, myogenesis and mitochondrial function. 1α,25(OH)_2_D3 was also associated with genes involved in skeletal muscle metabolism, protection against cell stress, and protein degradation. VDR forms a heterodimer with RXR in the presence of 1α,25(OH)_2_D3 to influence gene transcription, we observed a positive relationship between serum 25OHD3 and muscle *RXR* gene expression. Established VDR target genes such as *PPARD* and *PPARGC1A*, which are involved in protection against an adverse metabolic phenotype (obesity, insulin resistance) [31], were positively correlated with serum 25OHD3 in this study. This is also of interest as these targets are involved in mitochondrial biogenesis and energy metabolism, which vitamin D has recently been shown to regulate [32]. Serum vitamin D was positively correlated with *MYH2*, which is expressed in the fast type II muscle fibers that are preferentially lost in severe vitamin D deficiency.

Associations between serum vitamin D and genes encoding components of TGF-beta/myostatin signalling pathways were also seen with upregulation of *SMAD3*, *SMAD4*, *ACVR2A* and downregulation of *SMAD7*. This is consistent with previous data demonstrating modulation of SMADs by vitamin D, resulting in anti-proliferative effects and resolution of the rodent model of multiple sclerosis [33]. We also observed a positive correlation with *DDIT4*, expression of which has been shown to be up-regulated by active vitamin D *in vitro* resulting in suppression of mTOR activity with anti-proliferative effects [34]. This conflicts with results from a murine skeletal cell culture model, which showed that active vitamin D treatment increased protein synthesis via Akt-mTOR signalling [35]. Associations were also seen with targets involved in protein translation, such as *IGF1R*, and eukaryoric translation initiation factors, *eIF4BP1* and *EIF2B1*. A previous study in rat chondrocytes, showed that vitamin D induces upregulation of *IGF1R* expression, whilst IGF-1 stimulated *VDR* expression [36]. In view of the similar morphology between endocrine myopathies common mechanisms and the interplay between hormonal axes is an area of potential interest. The positive relationship between serum vitamin D and expression of several proteasomal subunits is unexpected, in that vitamin D has previously been demonstrated to antagonise ubiquitin-proteasomal dependent degradation of myofibrils *in vitro* [37]. No significant associations were observed between vitamin D and the muscle-specific ubiquitin ligases *MuRF1* and *MAFbx1* in this study however. Genes involved in cell stress response were also positively associated with serum vitamin D including *SOD1*, *CRYAB* and *HSP90AA*. The negative correlation between serum 25OHD3 and skeletal muscle *VDR* was unexpected; a previous study with a smaller number of participants reported no relationship [38], and another study reported upregulation of VDR in response to 1α,25(OH)_2_D3 [39]. Intriguingly, many of the gene expression associations with VDR (S1 Table) were the inverse of those observed with 25OHD3, including those involved in TGFβ/myostatin signalling, proteasomal and metabolic function. The precise explanation for this is unclear, but may include ligand-independent actions of VDR, as well as indirect effects of 25OHD3 on muscle mediated via responses in associated fat tissue.

Collectively these observations indicate that the link between vitamin D, muscle function and sarcopenia is more complex than originally thought, and cannot be defined simply through measurement of 25OHD3. General limitations of the study that should be taken into account include its cross-sectional nature, which precludes us from confirming causality or temporal effects. In view of the multiple parameters assessed, there is also the possibility of type 1 errors occurring in statistical analysis, however the positive findings are greater than would be expected assuming false discovery rate of 5%. Finally, baseline information on exercise potential was not recorded, although all participants were ambulatory, community-dwelling and not suffering from chronic diseases. Despite these caveats, data presented here highlight the limitations of studies where only conventional serum 25OHD3 measures are used. The distinct direct and indirect effects of 1α,25(OH)_2_D3 and 25OHD3 respectively on muscle function and gene expression suggest that multiple other factors must be considered when assessing the physiological impact of vitamin D deficiency or supplementation. Notably this includes measures of lean versus fat mass, and steroid metabolite profiles, but in future it will also be important to include a more comprehensive analysis of the metabolites that make up the vitamin D ‘metabolome’.

## Supporting information

S1 Table Bivariate correlations between serum 25OHD3 and gene expression in skeletal muscle. VDR = Vitamin D Receptor, RXRG = Retinoid X Receptor, Gamma; PPARD = Peroxisome Proliferator Activated Receptor Delta; PPARGC1A = Peroxisome Proliferator Activated Receptor Gamma, Coactivator 1 Alpha; HSP90AA1 = Heat Shock Protein 90kDa Alpha, Class A Member 1; CRYAB = Crystallin, Alpha B; SOD1 = Superoxide Dismutase 1; SMAD3 = SMAD Family Member 3; SMAD4 = SMAD Family Member 4; SMAD7 = SMAD Family Member 7; ACVR2A = Activin A Receptor, Type IIA; DDIT4 = DNA-Damage-Inducible Transcript 3; IGF1R = Insulin-Like Growth Factor 1 Receptor; PSMC4 = Proteasome (Prosome Macropain) 26S Subunit, ATPase 4; PSMC5 = Proteasome (Prosome Macropain) 26S Subunit, ATPase 5; PSMD11 = Proteasome (Prosome Macropain) 26S Subunit, Non-ATPase 11; PSMD12 = Proteasome (Prosome Macropain) 26S Subunit, Non-ATPase 12; PSMD14 = Proteasome (Prosome Macropain) 26S Subunit, Non-ATPase 14; eIF4BP1 = Eukaryotic Translation Initiation factor 4B Pseudogene 1; EIF2B1 = Eukaryotic Translation Initiation Factor 2B; GLUL = Glutamate-Ammonia Ligase; MYH2 = Myosin, Heavy Chain 2; Myogenin = Myogenic (Myogenic factor 4); CYCS = Cytochrome C, Somatic. Data are correlation coefficients (Spearman correlations (rho)).
(DOCX)
