# Supplementary material for: 25-hydroxyvitamin D3 and 1,25-dihydroxyvitamin D3 exert distinct effects on human skeletal muscle function and gene expression
Source: PLoS One. 2017 Feb 15;12(2):e0170665. doi: 10.1371/journal.pone.0170665 (PMC5310904; doi:10.1371/journal.pone.0170665)
Supplement: S1 Table — VDR = Vitamin D Receptor, RXRG = Retinoid X Receptor, Gamma; PPARD = Peroxisome Proliferator Activated Receptor Delta; PPARGC1A = Peroxisome Proliferator Activated Receptor Gamma, Coactivator 1 Alpha; HSP90AA1 = Heat Shock Protein 90kDa Alpha, Class A Member 1; CRYAB = Crystallin, Alpha B; SOD1 = Superoxide Dismutase 1; SMAD3 = SMAD Family Member 3; SMAD4 = SMAD Family Member 4; SMAD7 = SMAD Family Member 7; ACVR2A = Activin A Receptor, Type IIA; DDIT4 = DNA-Damage-Inducible Transcript 3; IGF1R = Insulin-Like Growth Factor 1 Receptor; PSMC4 = Proteasome (Prosome Macropain) 26S Subunit, ATPase 4; PSMC5 = Proteasome (Prosome Macropain) 26S Subunit, ATPase 5; PSMD11 = Proteasome (Prosome Macropain) 26S Subunit, Non-ATPase 11; PSMD12 = Proteasome (Prosome Macropain) 26S Subunit, Non-ATPase 12; PSMD14 = Proteasome (Prosome Macropain) 26S Subunit, Non-ATPase 14; eIF4BP1 = Eukaryotic Translation Initiation factor 4B Pseudogene 1; EIF2B1 = Eukaryotic Translation Initiation Factor 2B; GLUL = Glutamate-Ammonia Ligase; MYH2 = Myosin, Heavy Chain 2; Myogenin = Myogenic (Myogenic factor 4); CYCS = Cytochrome C, Somatic. Data are correlation coefficients (Spearman correlations (rho)). (DOCX) [file pone.0170665.s001.docx]

**S1 Table.**

| **Gene Function** | **Gene** | **rho** | **95% CI** | **p-value** |
| --- | --- | --- | --- | --- |
| **Nuclear Receptor** | VDR | -0.41 | -0.64- -0.10 | 0.008 |
| **Nuclear Receptor** | RXRG | 0.40 | 0.14-0.61 | 0.003 |
| **Nuclear Receptor/ transcription factor** | PPARD | 0.28 | 0.00-0.52 | 0.04 |
| **Transcriptional co-activator** | PPARGC1A | 0.35 | 0.08-0.57 | 0.01 |
| **Molecular Chaperone** | HSP90AA1 | 0.35 | 0.08-0.57 | 0.01 |
| **Molecular Chaperone** | CRYAB | 0.34 | 0.07-0.56 | 0.01 |
| **Cell Stress Response** | SOD1 | 0.29 | 0.02-0.53 | 0.03 |
| **Signal Transduction** | SMAD3 | 0.32 | 0.04-0.54 | 0.02 |
| **Signal Transduction** | SMAD4 | 0.28 | -0.00-0.51 | 0.04 |
| **Signal Transduction** | SMAD7 | -0.28 | -0.51-0.00 | 0.04 |
| **Signal Transduction** | ACVR2A | 0.27 | -0.00-0.51 | 0.046 |
| **Signal Transduction** | DDIT4 | 0.31 | 0.04-0.55 | 0.02 |
| **Signal Transduction** | IGF1R | 0.35 | 0.08-0.58 | 0.009 |
| **Proteasomal function** | PSMC4 | 0.27 | 0.00-0.51 | 0.048 |
| **Proteasomal function** | PSMC5 | 0.28 | 0.00-0.51 | 0.04 |
| **Proteasomal function** | PSMD11 | 0.28 | 0.00-0.51 | 0.04 |
| **Proteasomal function** | PSMD12 | 0.27 | 0.00-0.51 | 0.04 |
| **Proteasomal function** | PSMD14 | 0.33 | 0.06-0.55 | 0.02 |
| **Protein Translation** | eIF4BP1 | 0.37 | 0.10-0.59 | 0.006 |
| **Protein Translation** | EIF2B1 | 0.28 | 0.00-0.52 | 0.04 |
| **Amino Acid Metabolism** | GLUL | 0.35 | 0.08-0.57 | 0.01 |
| **Muscle Contraction** | MYH2 | 0.30 | 0.03-0.54 | 0.03 |
| **Myogenesis** | Myogenin | 0.27 | -0.00-0.51 | 0.04 |
| **Mitochondrial Function** | CYCS | 0.39 | 0.13-0.60 | 0.004 |
